# A data-driven method for surgeon-specific difficulty assessment in third molar extraction

**DOI:** 10.3389/fmed.2025.1654727

**Published:** 2025-11-07

**Authors:** Chun Kang, Ziyu Yan, Xiya Xiong, Zhilong Mi, Fei Wang, Binghui Guo, Binzhang Wu, Ziqiao Yin, Nianhui Cui

**Affiliations:** 1 School of Artificial Intelligence, Beijing Advanced Innovation Center for Future Blockchain and Privacy Computing, Beihang University, Beijing, China; 2 Key Laboratory of Mathematics, Informatics and Behavioral Semantics and State Key Laboratory of Complex & Critical Software Environment, Beihang University, Beijing, China; 3 Department of Oral and Maxillofacial Surgery, Peking University School and Hospital of Stomatology & National Center for Stomatology & National Clinical Research Center for Oral Diseases, National Engineering Research Center of Oral Biomaterials and Digital Medical Devices, Beijing, China; 4 First Clinical Division, Peking University School and Hospital of Stomatology & National Center for Stomatology & National Clinical Research Center for Oral Diseases & National Engineering Research Center of Oral Biomaterials and Digital Medical Devices, Beijing, China; 5 Hangzhou International Innovation Institute of Beihang University, Hangzhou, China

**Keywords:** impacted mandibular third molars, tooth extraction, machine learning, data-decoupling, difficulty assessment

## Abstract

**Background and objectives:**

The purpose of this study is to use a data-driven method to analyze the time taken by junior doctors to extract lower wisdom teeth and the factors affecting the difficulty of the procedure. It aims to reveal the distribution characteristics of difficulty factors at different stages of development, establish a mathematical model for procedural difficulty, evaluate the effectiveness of the existing difficulty scale, and provide difficulty indicators for the extraction training of impacted teeth for young doctors at different stages.

**Materials and methods:**

We collected surgical records of 419 cases of lower impacted wisdom teeth extraction completed by 9 residents. The difficulty index was based on a scale with 14 primary indicators and 37 secondary indicators. We proposed a data-driven method for surgeon-specific difficulty assessment (DDSS) of third molar extraction surgery. When assessing the surgical difficulty for a surgeon, the DDSS uses a method based on Lasso regression to classify the doctor as either a junior doctor who has completed grade 1 training or a novice doctor. It then calls upon the corresponding pre-trained model to conduct targeted difficulty prediction and provide key difficulty factors.

**Results:**

Our method achieved an accuracy of 80% and an AUC of 0.85 with SVM. The methods we proposed outperformed the methods without decoupling. The clustering analysis revealed that inexperienced surgeons are affected by a larger number of factors, while experienced surgeons are primarily influenced by four key factors: Crown resistance, impacted type, mouth opening, and gender. Learning curves indicated that surgeons typically become proficient after 8 months of practice.

**Conclusion:**

We propose a data-driven decoupling-prediction model, which improves the model’s performance in the task of assessing dental surgery difficulty. We also draw the learning curve of novice surgeons based on the data decoupling method we proposed. This provides a new perspective for surgical difficulty assessment and surgeon training, and offers a reliable conclusion.

## 1 Introduction

The extraction of impacted mandibular third molar is one of the most common procedures in oral and maxillofacial surgery. Given the anatomical variations and limited surgical visibility, these surgeries often present significant challenges (1), especially for residents. On the other hand, there is a large number novice of doctors in the field who need targeted training, and assigning them surgeries with inappropriate levels of difficulty poses safety risks (2–4). Therefore a reasonable evaluation can not only formulate more accurate surgical plans and optimize resource allocation, but also provide targeted training for novice doctors, accelerating their learning process.

Nowadays, there have been many rule—based methods to quantify the difficulty of extracting impacted mandibular third molars. These methods are established by experienced doctors who set relevant rules and apply them in clinical practice. The traditional Pell-Gregory classification is criticized for its unreliability in predicting extraction challenges (5). In recent years, scholars have introduced various new assessment methods that consider additional factors, however, those assessment still have limitations, such as oversimplified scoring systems and the lack of a theoretical basis for grading differences (6–8). To address these issues, the Delphi survey, a technique that facilitates group consensus through an iterative multistage process, was employed to develop a new scoring scale (9). This method involves soliciting, synthesizing, and refining expert opinions across multipole rounds, thereby avoiding mutual influence among experts and achieving more accurate and objective results (10). Chen et al. established a difficulty scoring scale for third molar extraction using the Delphi method.

However, these rule-driven methods overlook the growth potential of novice surgeons and fail to account for the complex and idiosyncratic situations of novice doctors. In recent years, some methods have tried to rebuild the assessment system from a data—driven perspective. Compared with rule—driven methods, data—driven methods can mine key information from data, and get a better performance (11–22). For example, Chen et al. (11) systematically reviewed the research progress of deep learning in caries detection, exploring the potential of this technology to improve diagnostic accuracy. Chen et al. developed a clinical decision support system that automatically generates diagrams for removable partial dentures based on textual design, simplifying the restoration process. Yamagami et al. (12) trained a decision—tree model to accurately assess the risk of postoperative infection and Van der Cruyssen et al. (13) established a postoperative risk—assessment system for third—molar surgery using the XGBoost model. These methods employ machine learning techniques, enabling the model to learn thoroughly in a data—driven manner for the corresponding tasks. They allow the model to learn the inherent patterns in the data at a relatively low cost, thus avoiding the introduction of a large number of manual rules. Drawing on these studies, we attempt to establish an assessment method that focuses on the growth potential of each surgeon by approaching from the perspective of data.

In summary, in addition to the previous rule-driven methods, this study proposes a new assessment method from a data-driven perspective. This approach attempts to address the issue of existing rule-driven methods that overlook the differences in the growth potential of novice surgeons. Specifically, the study introduces a data-driven decoupling-evaluation model. This model not only pays attention to the differences in the learning process of each surgeon, but also attempts to identify the common difficulty factors that affect surgeons at different stages.

## 2 Materials and methods

### 2.1 Study design and data sources

In this study, all data were obtained from the Department of Oral and Maxillofacial Surgery in Peking University School and Hospital of Stomatology, and the evaluation period was from December 24, 2020, to October 28, 2023. Our inclusion criteria were as follows: (1) The cases included were those treated entirely by one-year graduate resident in the Department of Oral and Maxillofacial Surgery, with the residents’ training period ranging from January to December, and who were assessed by senior physicians as capable of independently performing the extraction of impacted lower third molars. (2) The cases included were those of impacted lower third molar extractions, with complete preoperative imaging data and accurate records of surgical operation time during the procedure. (3) The patients included were aged 18–45 years and were able to fully cooperate with the surgical procedures. Our exclusion criteria were as follows: (1) The patient was missing the second molar on the side of the extraction. (2) The patient had significant dental anxiety or a pronounced gag reflex, making it impossible to perform the extraction under local anesthesia in an outpatient setting. (3) During the internship period, the resident had an interruption of more than 1 week or attended the outpatient clinic for less than 2 days per week. After screening, we collected surgical records of 419 cases of lower impacted wisdom teeth extraction completed by 9 residents, each case of data has 14 surgical features: Crown condition of second molar, Second molar looseness, Relationship of M3M and IAN, impacted type, Crown condition of M3M, Root number, Root Morphology, Root width, Crown resistance, Age, Mouth opening, BMI, and gender.

To ensure data integrity, all 14 primary and 37 secondary indicators were checked for completeness. Missing values accounted for less than 0.05% of the entire dataset. For these rare cases, group-wise mean imputation was applied. We further verified that imputing these values did not significantly affect model performance (AUC change < 0.01). This preprocessing procedure ensured the robustness and reproducibility of subsequent analyses.

All analyses were conducted using Python version 3.12.3 with the scikit-learn package (version 1.5.2). Descriptive statistics were expressed as mean ± standard deviation for continuous variables and frequency (percentage) for categorical variables. Independent sample *t*-tests were applied to compare operative times between groups, with a significance level of *p* ≤ 0.05. For machine learning models, performance was evaluated using accuracy, sensitivity, specificity, F1-score, and the area under the receiver operating characteristic curve (AUC). Five-fold cross-validation was conducted to ensure the robustness of the results.

The purpose of this study was to evaluate surgical difficulty, with the surgeons themselves as the research subjects. No patient treatment interventions or follow-ups were involved. The core data consisted of operative time and related surgical factors recorded independently by assistants during the procedures, focusing solely on the assessment of operative difficulty. At no point were patient identifiers collected, recorded, or disseminated, and the data could not be linked to any specific patient. Therefore, no patient interests were affected, and ethical approval was not required. Specifically, operative times were documented by assistants during the procedures, along with the relevant case information, which was then compiled and delivered to the first author for statistical analysis. The operators were affiliated with Peking University School and Hospital of Stomatology, while the first author was affiliated with Beihang University and had no access to patient identity information.

### 2.2 Overview of DDSS

**Figure 1:**
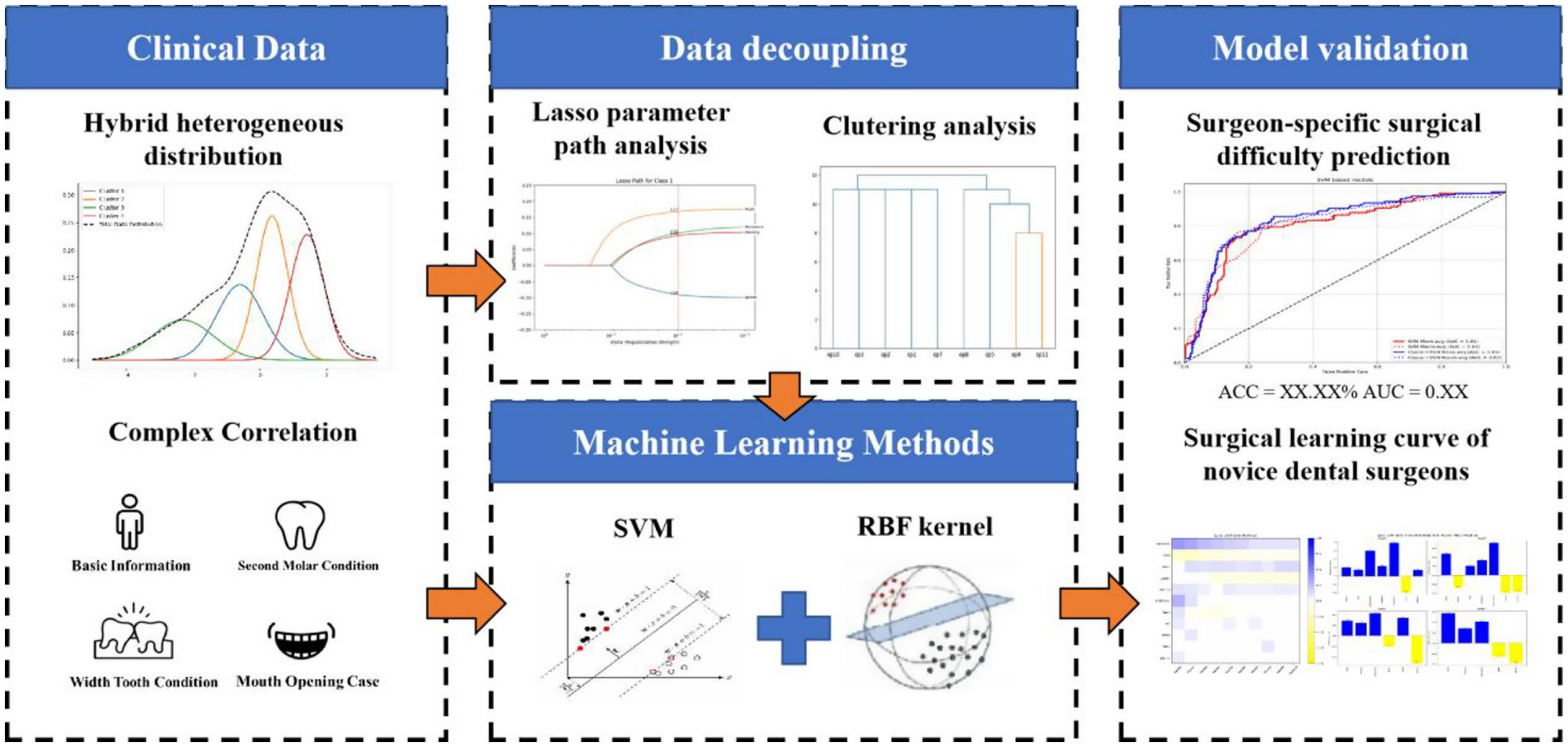
Overview of DDSS.

We propose a decoupling-prediction model to classify and predict different types of surgeons, with the overall workflow as follows: first, we calculate the surgical preference vector for each surgeon based on the Lasso regression (23–25) method and measure the similarity between vectors using the Levenshtein distance. Subsequently, we use hierarchical clustering to divide the surgeons into two categories and train a machine learning model for each category (26). During the training process, we split the data for each surgeon into training and testing sets at a ratio of 4:1. The overall training set is composed of the training data from all surgeons (see Figure 1).

### 2.3 Construction of feature vectors

We used the Lasso regression method to determine the feature order based on the sequence in which features transition from zero to non-zero as the regularization parameter lambda decreases. Specifically, we plot the Lasso curve for each doctor and record the number of times the coefficients of all features change from zero to non-zero as the regularization parameter lambda decreases from positive infinity to zero. By doing so, we constructed unique feature vectors for the surgical features of each surgeon. In this study, we used Python 3.12.3 and called the scikit-learn library (version 1.5.2) to implement this process.

### 2.4 Data decoupling

After obtaining the surgical vectors for each surgeon, we used the Levenshtein distance to measure the surgical similarity between surgeons and applied hierarchical clustering to divide all surgeons into two major categories. The Levenshtein distance is a measure of the minimum number of single-character edits (insertions, deletions, or substitutions) required to change one string into another, commonly used to quantify the similarity between two sequences. Hierarchical clustering is a method that builds nested clusters by successively merging or splitting existing clusters based on distance metrics, resulting in a tree-like structure called a dendrogram. Through these method, we were able to identify surgeons with similar surgical preferences. In this study, we used Python 3.12.3 and called the scikit-learn library (version 1.5.2) to implement this process.

### 2.5 Machine learning prediction

We designed Support Vector Machine (SVM) to learn from each category of surgeons (27–33). To enhance the SVM’s ability to understand complex data, we introduced the radial basis kernel function. We optimized the penalty parameter (C) and kernel parameters using grid search combined with five-fold cross-validation. The optimal configuration was C = 100 with a radial basis kernel, which achieved the highest validation AUC and was subsequently adopted for testing. The same parameter-tuning strategy was applied to Random Forest and XGBoost for fair comparison. Besides, we also selected XGBoost and Random Forest for comparison (17–22). Through this approach, we can specifically learn the surgical features of surgeons with similar surgical preferences. In the experiment, we used Python 3.12.3 and called the scikit-learn library (version 1.5.2) to implement these process.

## 3 Results

In summary, the main findings of this study were that most extractions were completed within 20 min, clustering analysis effectively distinguished inexperienced from experienced residents, and the proposed decoupling SVM model achieved the highest predictive performance with an accuracy of 80% and an AUC of 0.85. In addition, learning curve analysis suggested that residents typically required about 8 months of practice to become proficient, with crown resistance, impaction type, mouth opening, and gender identified as the key factors influencing surgical difficulty.

### 3.1 Basic statistical analysis

We conducted a basic statistical analysis of the 419 surgical records, and the results are shown in Table 1. Based on the distribution of surgical duration, we found that 25.24% of surgeries were completed within 10 min (600 s), while 76.21% were completed within 20 min (1,200 s). For ease of calculation, we set the first quartile at 600 s and the third quartile at 1200 s. Combining clinical expertise, we classified cases into three difficulty levels: Class 1: Surgeries completed in under 10 min. Class 2: Surgeries taking 10 to 20 min. Class 3: Surgeries exceeding 20 min. Additionally, to account for variations among different surgeons, we first normalized the surgical duration for each surgeon based on their recorded operation times. In this experiment, we divided the dataset into a training set and a test set in a 4:1 ratio.

**Table 1:** Baseline surgical characteristics.

Primary indicators	Abbreviation	Secondary indicators	Proportions
(1) Crown condition of second molar	SMC	Prothesis	0.3%
		Distal tooth defect or filling	9.6%
		Other situations	90.0%
(2) Second molar looseness	Stability	Loose	5%
		Not loose	94.9%
(3) Relationship of M3M and IAN	IAN	Uncontacted	67.3%
		Overlap	10.1%
		Intrude	22.2%
(4) Impacted type (winter classification)	Angle	Distal	8.2%
		Vertical and mesial (located above the contour point of second molar)	26.6%
		Mesial (located below the contour point of second molar)	26.3%
		Horizontal	34.3%
		Inverted	0.4%
		Buccal	1.2%
		Lingual	2.4%
(5) Depth (Pell & Gregory classification)	Depth	High	49.3%
		Medium	44.5%
		Low	5.8%
(6) Crown condition of M3M	Crown	Tooth defect (non-mesial defect more than 1/2)	7.9%
		Unbroken or small decay	91.7%
(7) Root number	Roots	Two	52.5%
		Three or more	2.1%
		One or fusion	45%
(8) Root morphology	Morphology	Complete development	3.8%
		Incomplete development	7.2%
		Bending in opposite direction or to mesial	1.4%
		Bending in two or more different direction	2.1%
		Enlargement in apical site	84.9%
(9) Root width a: cervical width b: maximum root width	Width	a>b or a ≈ b	84.0%
		a<b	15.7%
(10) Crown resistance	Resistance	Complete eruption	8.9%
		Soft tissue coverage	49.1%
		Partial bone coverage	40.4%
		Complete bone coverage	1.2%
(11) Age	Age	0–25	30.2%
		25–35	54.7%
		>35	14.7%
(12) Mouth opening	Opening	Normal	98.5%
		Limited mouth opening	1.2%
(13) BMI	BMI	<18.5	11.4%
		18.5–25	69.6%
		25–30	15.0%
		30–35	3.3%
		>35	0.4%
(14) Gender	Gender	Male	40.5%
		Female	59.4%

The specific surgical qualifications of each surgeon are shown in Table 2, which presents the surgical experience, average surgical time and standard deviation, longest surgical duration, and shortest surgical duration of each surgeon (Unit: seconds).

**Table 2:** The specific surgical qualifications of each surgeon.

Surgeon	Surgical experience	Average surgical time with std (second)	Longest surgery duration (second)	The shortest surgery duration (second)
Surgeon 1	10 months	914 (±435)	2,700	40
Surgeon 2	10 months	1,062 (±438)	2,100	180
Surgeon 3	10 months	759 (±348)	2,400	240
Surgeon 4	6 months	917 (±342)	1,500	480
Surgeon 5	6 months	1,160 (±634)	2,700	180
Surgeon 6	1 months	1,585 (±577)	2,520	1,080
Surgeon 7	4 months	1,091 (±415)	1980	420
Surgeon 8	5 months	1,075 (±490)	2,100	300
Surgeon 9	1 months	949 (±446)	1,560	240

### 3.2 The performance of different clustering algorithms and machine learning methods in the classification of oral surgery difficulty

**Figure 2:**
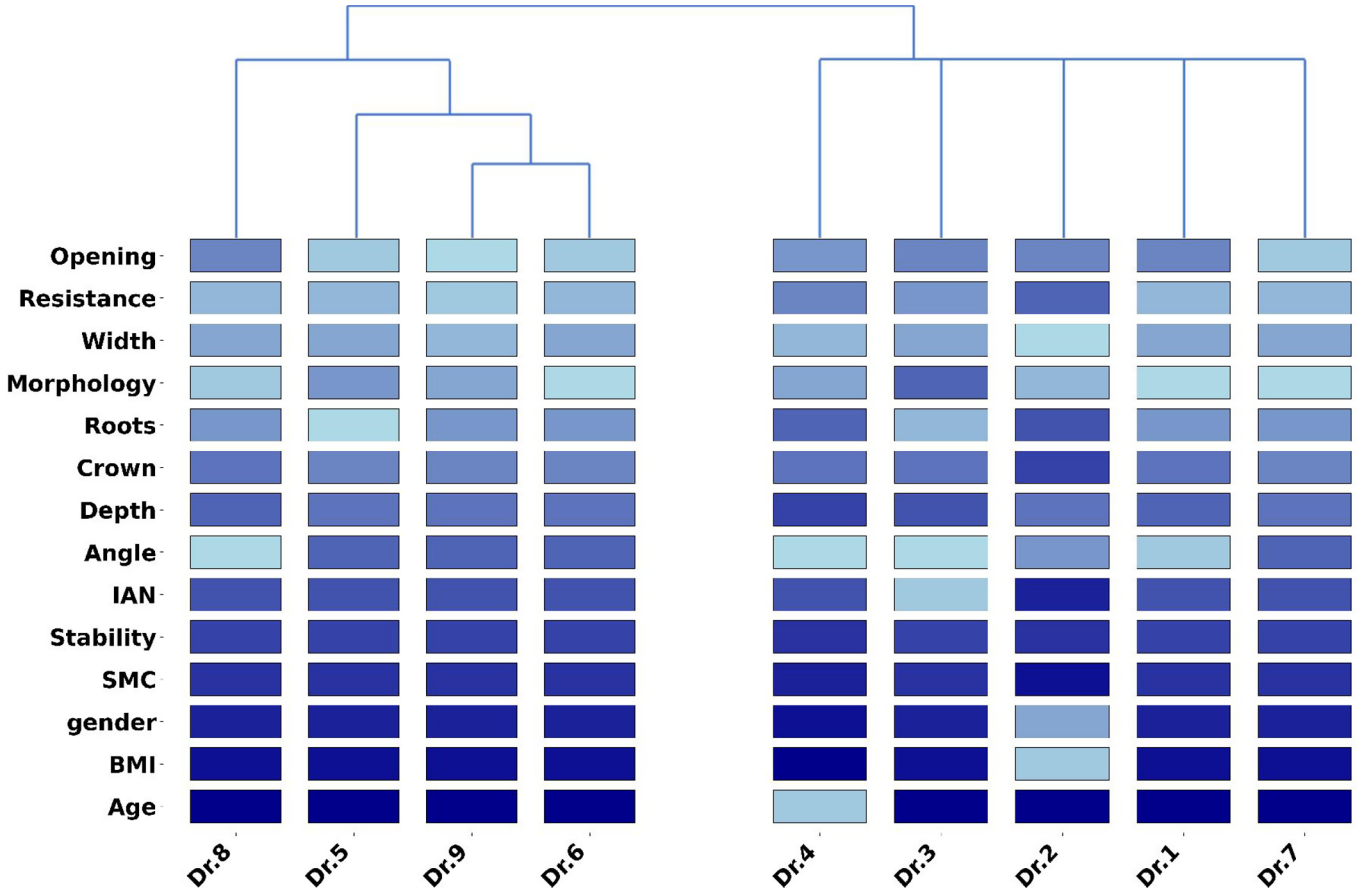
This figure shows the surgical feature labels corresponding to different surgeons and the clustering results for all surgeons. Among them, surgeons 1, 2, 3, 4, and 7 are grouped into the first category, while surgeons 5, 6, 8, and 9 are classified into the second category.

First, we calculated the surgical preference sequence labels for all the surgeons based on the LASSO parameter trajectory method we proposed, as shown on the left side of Figure 2. Each label reflects the surgical preferences of the corresponding surgeon to some extent. Subsequently, we applied hierarchical clustering to these surgical preference sequence labels, using the Levenshtein distance to measure the similarity between sequences, as shown on the right side of Figure 2. The experimental results indicate that the surgical behaviors of surgeons no. 1, 2, 3, 4, and 7 are relatively similar, while those of surgeons no. 5, 6, 8, and 9 are relatively similar. Therefore, we conclude that the surgeons can be divided into two major groups: the first group includes surgeons no. 1, 2, 3, 4, and 7, and the second group includes surgeons no. 5, 6, 8, and 9.

**Figure 3:**
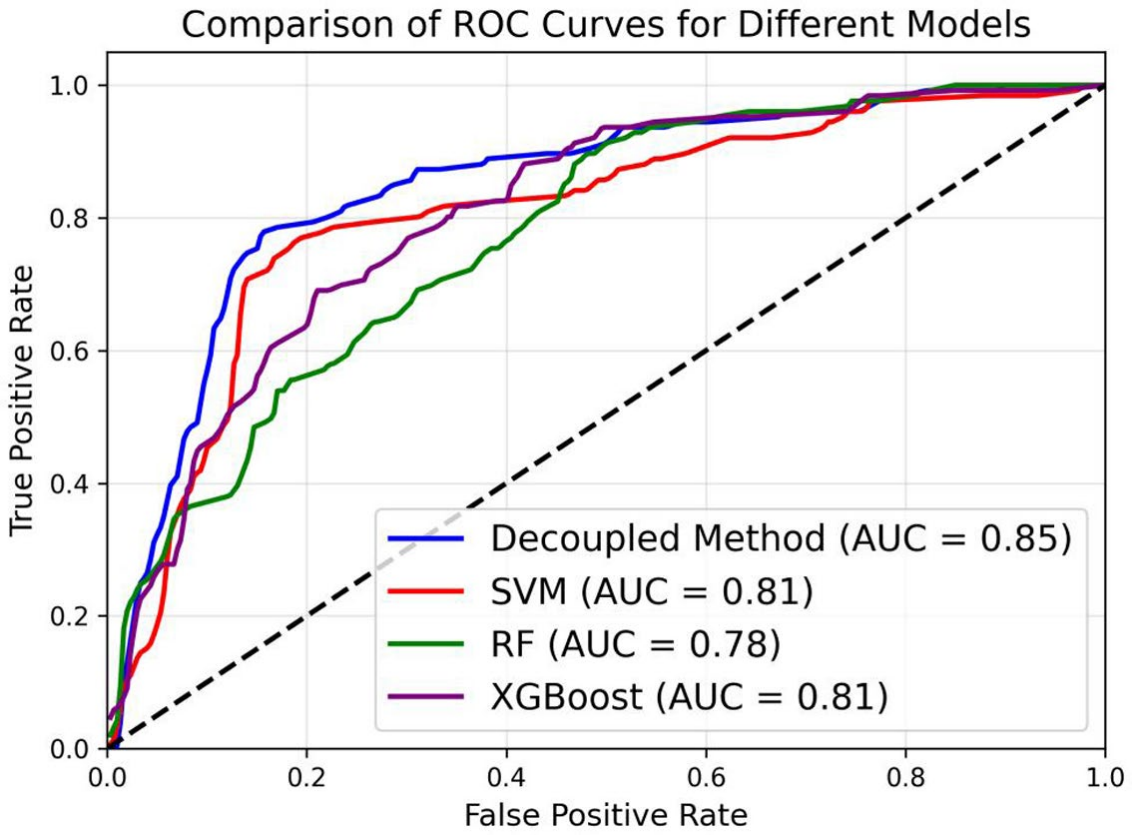
Macro-average ROC curve of three machine learning models and SVM with Lasso-based clustering method in the task of predicting the difficulty of oral surgery.

After obtaining the clustering results, we trained a specific model for each cluster using three machine learning algorithms. During the testing phase, we first determined the closest cluster for each test sample and then used the model trained on that cluster for prediction. In addition to our proposed method of hierarchical clustering based on the label vectors obtained from the LASSO trajectory, we also applied the commonly used GMM algorithm in the medical field for comparison. The results are shown in Table 3. The experimental results demonstrate that the approach of clustering the data before model training outperforms the method without clustering. The performance improved by at least 2% when clustering was applied compared to when it was not. In addition, after adopting the decoupling algorithm, the AUC metric reached 0.85, which is 4% than higher without the decoupling algorithm. Moreover, our proposed clustering method achieved results of 72 and 68% with Random Forest and XGBoost, respectively, which are on par with the results obtained using the GMM algorithm. Notably, our clustering method achieved the best performance with SVM, reaching an accuracy of 80%, while the method based on GMM clustering only achieved an accuracy of 78%. On the other hand, as shown in Figure 3, our model achieves the best performance in the macro-average ROC curve, with an AUC of 0.85. This indicates that the model delivers optimal average performance across all categories, demonstrating strong robustness.

**Table 3:** Results of different clustering methods and three machine learning models in the task of predicting the difficulty of oral surgery.

Clustering algorithm	SVM	RF	XGBoost
Lasso clustering	80%	72%	68%
GMM clustering	78%	72%	68%
Without clustering	75%	70%	66%

### 3.3 Research on classification criteria and the impact of different features

**Figure 4:**
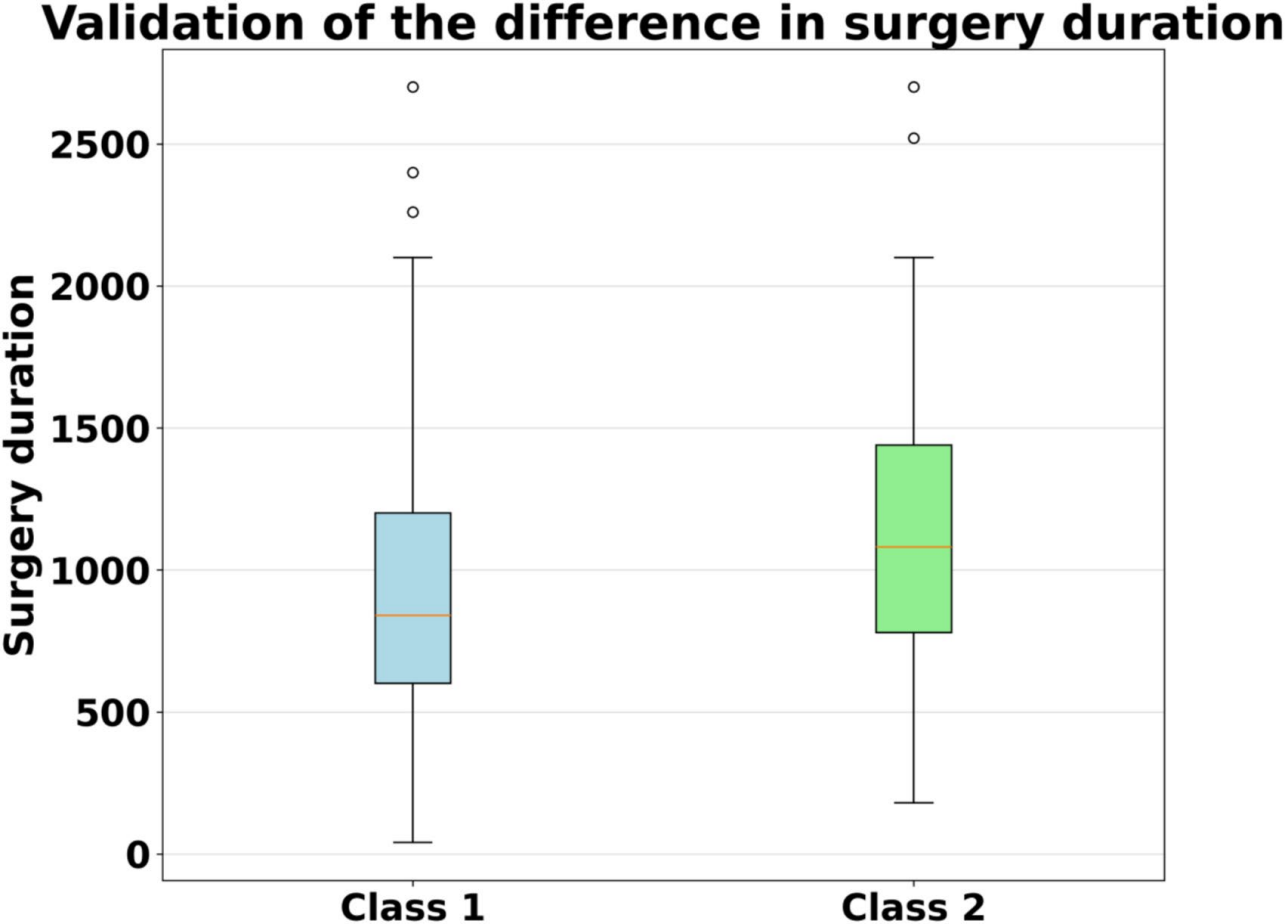
Boxplot of surgical duration for the two groups of surgeons.

In order to investigate the rationale behind our proposed clustering algorithm, we conducted an analysis using the student *t*-test and the test results are shown in Figure 4. The results indicate a significant difference in the duration of surgery between the two groups of surgeons, with the second group of surgeons having a significantly longer surgical duration than the first group. Upon comparison, we found that the first group of surgeons had a longer tenure in the department, with an average experiences of 8 months, while the second group had an average tenure of only 3.25 months.

Therefore, we conclude that the first group of surgeons are more proficient in surgical operations, resulting in shorter surgical times. In contrast, the second group of surgeons are less experienced, leading to longer surgical times. Therefore, we infer that the first group of surgeons are those who have completed the grade 1 training, while the second group consists of novice surgeons.

**Figure 5:**
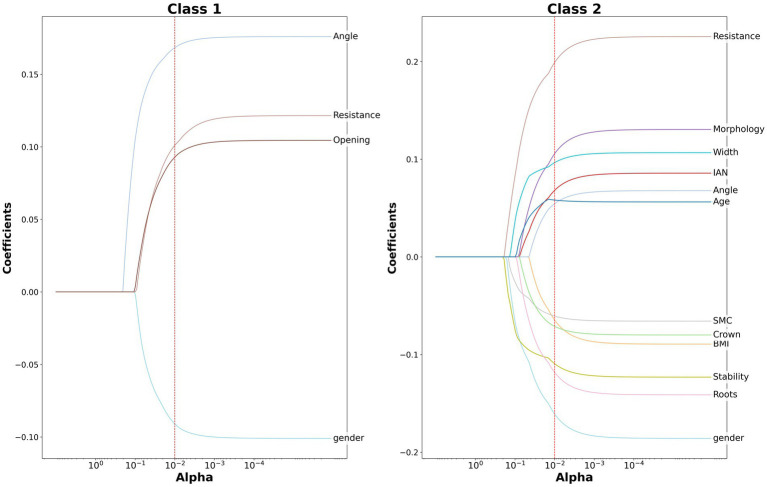
Lasso parameter trajectory curves for the two groups of surgeons.

We applied the Lasso trajectory method to observe the differences between the two groups of surgeons in terms of various features. Specifically, we drew Lasso parameter trajectory curves for each group of surgeons separately and retained the features with absolute values of parameters greater than or equal to 0.05 after truncation at *λ* = 0.01. These features are considered to significantly affect the difficulty of surgery. The results are shown in Figure 5. For the first group of surgeons, only four features were retained: Crown resistance, impacted type, gender and mouth opening. Among them, the coefficient of the feature impacted type is 0.17, the coefficient of Crown resistance is 0.10, the coefficient of mouth opening is 0.09, and the coefficient of gender is −0.09. In contrast, for the second group of surgeons, all factors were retained.

### 3.4 Learning curve of surgeons after joining the department by monthly division

**Figure 6:**
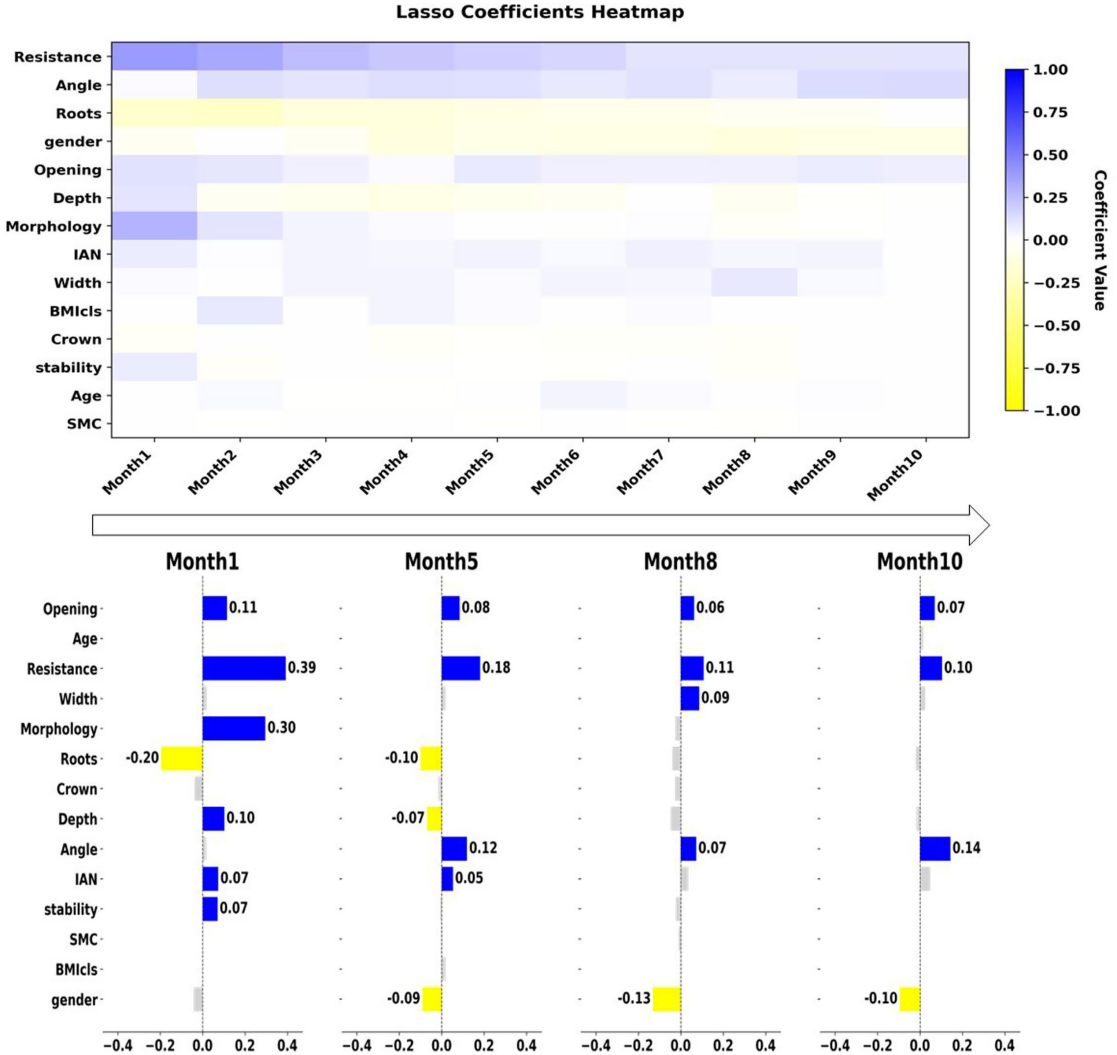
The upper panel shows the learning curve of surgeons after joining the department, plotted on a monthly basis. The lower panel shows the retained features and their corresponding parameters at 1, 5, 8, and 10 months.

To further explore the growth process of the surgeons, we have specifically drawn learning curves to better observe the growth of surgeons after joining the department. These learning curves reflect the changes in surgical performance on a monthly basis after joining the department. For this analysis, we obtained the records of all surgeons for their first 10 months, divided them by month, and drew Lasso parameter trajectories based on the corresponding surgical records. To more intuitively demonstrate the growth process of the surgeons, we presented the coefficients of each surgical feature at the 1st, 5th, 8th, and 10th months. The results are shown in Figure 6.

Compared with the Delphi method, we found that all the factors mentioned in the Delphi method are reflected in our study, and different factors show different performances at different stages. The Delphi method considers the four factors—Depth, impacted type, Relationship of M3M and IAN, and mouth opening—to be more critical in the surgical procedure, which slightly differs from our conclusion. In fact, these factors are distributed across different periods and have varying impacts on the surgical difficulty during different stages. For example, the “Depth” factor is a highly significant influencing factor in the early stages. However, after 6 months, the impact of this factor diminishes to some extent. Similarly, the “Relationship of M3M and IAN” factor has a very significant impact on the surgery in the first 6 months, but its impact diminishes after 7 months. In contrast, we found that Factor “mouth opening” did not change significantly over the 10 month period and remained a factor with a substantial impact on the surgery. This phenomenon also applies to the Crown resistance factor, with the only difference being that this factor did not have a significant impact on the surgeon in the first month. However, starting from the second month, it became a factor that influences the surgery. Overall, the learning curve we proposed shows the evolution of each feature over time.

Our study, for the first time in comparison with these rule-based methods, emphasizes the growth curve of novice surgeons. As can be seen from Figure 6, the learning curves of the surgeons’ surgical features initially diverge but eventually converge on a few factors. This indicates that the surgeons, who are initially sensitive to all feature factors, become sensitive only to a few factors over time, demonstrating the growth process of the surgeons. It can be seen that in the first month after joining the department, surgeons are sensitive to the vast majority of features. After the fifth month, only these seven factors have a significant impact on the surgery. After the eighth month, all factors except these 5—crown resistance, impacted type, Root width, gender, and mouth opening—become insignificant. And by the ninth and tenth months, only four factors have a significant impact on the surgery, which are impaction resistance of wisdom teeth, impacted type, gender and mouth opening. The process reflects that for novice surgeons, the influencing factors evolve from being complex and variable to eventually converging into core four factors during the learning process.

## 4 Discussion

The extraction of impacted mandibular third molar is one of the most common procedures in oral and maxillofacial surgery. Given the anatomical variations and limited surgical visibility, these surgeries often present significant challenges, especially for residents. On the other hand, there is a large number novice of doctors in the field who need targeted training, and assigning them surgeries with inappropriate levels of difficulty poses safety risks. Therefore, we urgently need a surgical assessment system tailored for novice doctors. A reasonable assessment can not only formulate more accurate surgical plans and optimize resource allocation, but also provide targeted training for novice doctors, accelerating their learning process.

Previous studies have proposed various approaches to evaluate surgical difficulty. Rule-based systems (5–10), such as the Pell–Gregory classification and the Delphi method, provide standardized frameworks but rely heavily on expert consensus and often fail to reflect the dynamic learning process of novice surgeons. With the rise of artificial intelligence, data-driven approaches have become increasingly popular. For example, Yoo et al. (34) used convolutional neural networks on panoramic radiographs to predict third molar extraction difficulty, while Karkehabadi et al. (35) applied deep learning to periapical radiographs to classify endodontic case complexity, both achieving high accuracy. These data-driven studies emphasize anatomical complexity but generally overlook the role of operator performance. Beyond dentistry, radiomics and machine learning models have been successfully applied in cancer diagnosis, genetic prediction, and clinical risk assessment (17–22). Such work demonstrates the broad potential of data-driven methods to support medical decision-making. Our study shares this data-driven philosophy but differs in focus. Instead of relying solely on anatomical or imaging features, we incorporated operative time and surgeon-specific data to capture both case complexity and the learning curve of residents. This approach revealed that residents typically required about 8 months to achieve proficiency, with crown resistance, impaction type, mouth opening, and gender identified as the most influential factors. By integrating surgeon performance into difficulty assessment, our framework provides an objective and practical tool for surgical education and competency evaluation.

In this study, we propose a data-driven method to address those issues. We proposed a data-driven method for surgeon-specific difficulty assessment (DDSS) of third molar extraction surgery, which is highly interpretable and can provide rational explanations for evaluation decisions. The DDSS method comprises a decoupler and a predictor. Specifically, the decoupler is responsible for categorizing the doctor into an appropriate group and providing targeted difficulty-influencing factors, while the predictor is in charge of offering a difficulty prediction result to ensure that the doctor is suitable for the particular surgery. Through this approach, we divided surgeons into two major categories. It has been verified that the first category of surgeons belongs to surgeons who have completed the grade 1 training, while the second category belongs to novice surgeons different decoupling algorithms and different machine learning models. The experimental results showed that clustering the data before training machine learning models yielded better performance compared to not using any clustering method. Moreover, our proposed decoupling method outperformed or matched the GMM method, which was the best result among all experiments. These findings demonstrate the superiority of our proposed method.

To explore the learning curve of novice surgeons, we plotted the learning curve of novice doctors from the beginning to the completion of the first year of training, and the results are shown in the upper part of Figure 6. Compared with traditional methods such as the Delphi method, for the first time we have presented the changing trend of difficulty factors from the perspective of the growth process. We have also precisely located these factors in terms of time to show the changes in the learning process of novice surgeons. For example, factors such as depth and root number will significantly affect the surgical difficulty in the first month. However, as time goes on, their impact on the surgery gradually decreases and by the eighth month, they no longer have a significant impact on the surgical difficulty. This indicates that a doctor who has completed the grade 1 training is already able to skillfully handle these factors. Nevertheless, the mouth opening factor will have a certain impact on the surgical difficulty from the first month to the tenth month. But in the first month, compared with depth and root number, this factor does not show a significant impact on the surgery. Meanwhile, impacted type factor does not have a significant impact on the surgery even in the first month. However, as doctors grow, this difficulty factor gradually becomes more significant. This shows that with the doctors’ in-depth learning, some factors will be skillfully handled by the doctors, while the importance of some factors gradually emerges. Overall, the learning curve reflects the learning process of the surgeons. In the first month after joining the department, the vast majority of factors had a significant impact on the surgical difficulty. After 8 months, only Crown resistance, impacted type, gender and mouth opening had a significant impact on the surgery. This indicates that 8 months is a crucial point for novice surgeons to reach the level of grade 1 training. Meanwhile, it also demonstrates that these four factors have a significant impact on the surgical difficulty, and they should be given particular attention when designing surgical plans. This conclusion provides a solid theoretical basis for surgeon training and fills the gap in the relevant field.

**Figure 7:**
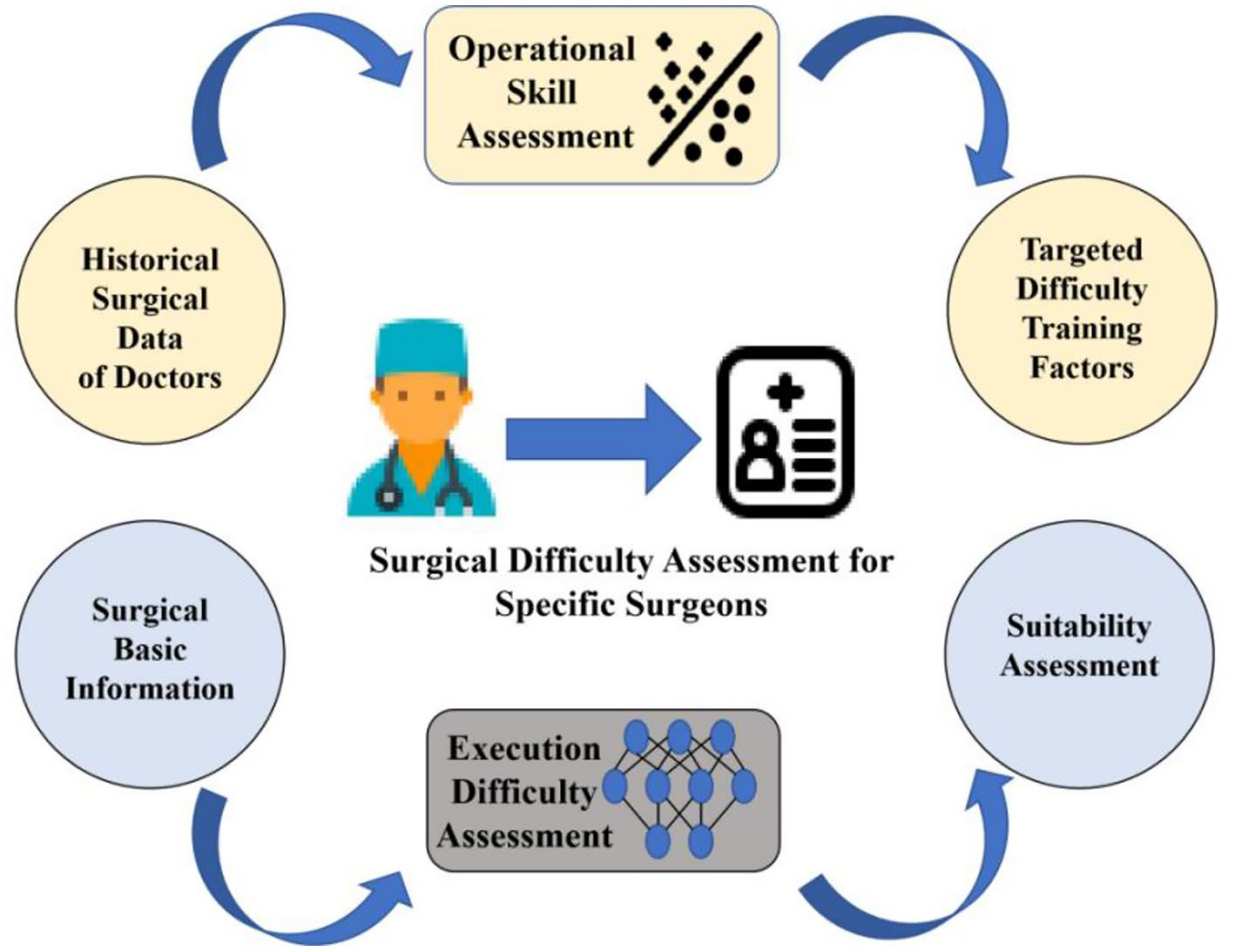
Model applications.

In practical application scenarios, by employing the DDSS method we proposed, we can conduct targeted assessments for doctors who have a limited number of third molar extraction surgery samples, as shown in Figure 7. Specifically, when we need to evaluate the difficulty of a surgery for a doctor with a small amount of historical surgical data, the decoupler of DDSS will carry out an operational level assessment, while the classifier will conduct a difficulty assessment of execution. They will, respectively, identify targeted difficulty factors and provide a difficulty assessment for the current surgery. This method is not only suitable for assessing novice surgeons to provide targeted training but is also applicable to other surgeons with only a few surgical samples, such as newly arrived surgeons, thereby enabling more accurate predictions.

Beyond individual case assessment, the DDSS framework can be seamlessly integrated into clinical education and training systems. For instance, it can be used as a digital evaluation module in resident training programs to automatically track surgeons’ learning curves, identify key difficulty factors affecting each stage of skill development, and generate personalized feedback reports. By continuously updating the model with new surgical records, institutions can employ DDSS to assign cases of appropriate complexity, optimize supervision strategies, and ensure both patient safety and educational effectiveness. We are currently developing a prototype training-assessment platform based on this framework for multi-center validation.

Despite its promising results, this study has several limitations. First, it was conducted at a single institution, which may limit the generalizability of the findings to other training environments. Future work will expand the dataset through multi-center collaboration to enhance model robustness and external validity. Second, although all surgical indicators were recorded objectively by assistants during operations, potential recording bias cannot be completely excluded. Finally, the sample size of 419 cases, while sufficient for model development, can be further increased to support deeper model architectures such as neural networks. Addressing these issues will form the next phase of our research.

In future work, we will establish a multi-center collaboration to enlarge the dataset and craft more diverse evaluation metrics, thereby boosting model robustness and external validity. We will also explore additional architectures such as CNNs and Transformers. Ultimately, we will extend our data-driven assessment pipeline beyond dentistry to a wider range of medical disciplines.

## 5 Conclusion

This study proposed a data-driven framework for evaluating surgical difficulty in mandibular third molar extractions, focusing on operative time and surgeon performance rather than patient outcomes. The decoupling SVM model demonstrated the best predictive performance, achieving an accuracy of 80% and an AUC of 0.85. Clustering analysis distinguished inexperienced from experienced residents, showing that inexperienced surgeons were influenced by multiple factors, while experienced surgeons were mainly affected by crown resistance, impaction type, mouth opening, and gender. Learning curve analysis further revealed that residents generally required approximately 8 months of practice to become proficient. These findings suggest that our approach not only provides an objective tool for surgical difficulty assessment but also offers practical insights into training evaluation and curriculum design for dental education.

## Data Availability

The data analyzed in this study is subject to the following licenses/restrictions: further inquiries can be directed to the corresponding author. Requests to access these datasets should be directed to Nianhui Cui, drcuinianhui@163.com.
